# Universal health care delivery mitigates socioeconomic-related risk for adverse outcomes in hospitalised patients: Lessons from the COVID-19 pandemic in Australia

**DOI:** 10.1371/journal.pone.0322780

**Published:** 2025-05-14

**Authors:** Fahimeh Faqihi, Rita Perri, Jimmy Chien, Jin-Gun Cho, Stephen Milne, Shopna Bag, Nicole Gilroy, John R. Wheatley, Kristina Kairaitis

**Affiliations:** 1 Department of Respiratory and Sleep Medicine, Westmead Hospital, Sydney, New South Wales, Australia; 2 Sydney Medical School, Faculty of Medicine and Health, The University of Sydney, Sydney, New South Wales, Australia; 3 Ludwig Engel Centre for Respiratory Research, The Westmead Institute for Medical Research, Sydney, New South Wales, Australia; 4 Centre for Population Health, Western Sydney Local Health District, Sydney, New South Wales, Australia; 5 Sydney School of Public Health, Faculty of Medicine and Health, The University of Sydney, New South Wales, Australia; 6 Department of Infectious Diseases, Westmead Hospital, Sydney, New South Wales, Australia; CHUV: Centre Hospitalier Universitaire Vaudois, SWITZERLAND

## Abstract

**Background:**

Internationally, socioeconomic disadvantage is related to severe outcomes of COVID-19. We investigated the impact of socioeconomic disadvantage on infection rates, hospitalisation, and in-hospital outcomes for COVID-19 with standardised medical care.

**Methods:**

This retrospective cross-sectional study included SARS-CoV-2 PCR-confirmed patients ≥18 years, admitted to a major public hospital between January 2020 and December 2021. Severe COVID-19 outcomes were defined by a composite outcome of in-hospital death or other critical complications. A generalised linear regression model of demographic features, co-existing conditions, and socioeconomic status was used to determine the risks of the composite outcome.

**Results:**

Of 797,343 individuals ≥18 years in the health district, 50,906 (6.4%) were PCR-positive, and 1,962 were hospitalised. Compared with the whole health district population, infected individuals were younger (median [interquartile range] age 35 [25–48] years vs 42 [31–58] years) and from areas with the greatest socioeconomic disadvantage (34.4% vs 20%; both p < 0.0001). Hospitalised patients were older, with more females compared to the PCR-positive group (46 years [33–61], 53.5%, respectively; p < 0.001), and 51.2% were from postcodes with greatest socioeconomic disadvantage (p < 0.0001). The composite outcome occurred in 11.5%, with an in-hospital mortality of 3.8%. Higher risk of the composite outcome was observed in males (OR 1.72, 95% CI [1.26–2.42], p < 0.001), patients aged ≥ 65 years (OR 6.96, [3.3–14.6], p < 0.001), those with ≥ 4 comorbidities (OR 2.67, [1.54–4.63], p < 0.001), and unvaccinated patients (OR 1.57, [1.05–2.38], p < 0.05). The risk of composite outcome did not increase with socioeconomic disadvantage (OR 0.97, [0.68, 1.42], p = 0.64).

**Conclusion:**

In the absence of capacity restraints, socioeconomic disadvantage was not associated with severe in-hospital outcomes in a well-resourced care environment despite increased rates of infection and hospitalisation. This highlights the impact of universally accessible, standardised, protocolised, high-quality in-hospital care in reducing the risk of adverse in-hospital outcomes in socioeconomically disadvantaged patients.

## Introduction

The COVID-19 pandemic has highlighted the impact of social determinants (economic stability, education access and quality, health care access and quality, neighbourhood and built environment, social and community context) on health outcomes [1,2]. Internationally, socially and economically disadvantaged individuals were more likely to be infected with SARS-CoV-2 [3,4], more likely to be hospitalised [5], more likely to be admitted to intensive care units, and have fatal outcomes [3,6,7]. This experience was global and reported in the United States [3,6], United Kingdom [5], Canada [8], Sweden [9], Germany [4], Japan [10] and Mexico [7].

In Australia, aligned with international reports, higher rates of SARS-CoV-2 infection in geographic areas associated with greater socioeconomic disadvantage were recorded in Victoria in 2020 [11] and Queensland between 2020 and 2022 [12]. Additionally, the Australian age-standardised COVID-19 mortality was reported to be higher for the most socioeconomically disadvantaged groups [13]. A population-wide study in NSW between January and October 2020 reported hospitalisation, intensive care admission, and mortality were more likely in those with the greatest socioeconomic disadvantage [14].

In 2020–2021, the population of a local health district was at the centre of the COVID-19 pandemic in Australia. This district has a population of over 1 million, 49.9% of whom were born overseas, 54.3% speak a language other than English at home, and 1.5% identify as Aboriginal or Torres Strait Islander [15]. In addition to cultural and ethnic diversity, this health district has a wide distribution of socioeconomic status ranging from relative wealth to significant disadvantage. Hospitalisation for COVID-19 during this time was predominantly at the largest public, specialised, tertiary teaching hospital within the local health district, with very few admissions elsewhere. This provided a unique opportunity to study the effects of socioeconomic disadvantage on health outcomes in a once-in-a-generation public health emergency.

This study aimed to use the unique diversity of the local health district and predominantly single hospital admission policy to examine the impact of socioeconomic disadvantage on the SARS-CoV-2 infection rates, hospitalisation rates, and severe in-hospital outcomes, including mortality, prior to widespread vaccination. In view of the worldwide experience, we hypothesised that patients from areas with greater socioeconomic disadvantage were likely to experience worse clinical outcomes.

## Methods

### Study design

We performed a retrospective review of hospital records for all SARS-CoV-2 PCR-positive patients admitted to a major public hospital between January 23, 2020 (the first COVID-19 patient) and December 31, 2021, compared with all SARS-CoV-2 PCR-positive individuals within the local health district and the whole health district census population (2021). The study was approved by the Western Sydney Local Health District Human Research Ethics Committee (HREC 2021/ETH12176). The data were accessed on January 19, 2022, and all data were de-identified before analysis. No authors have access to data that could identify individuals during or after data collection.

### Data collection

#### Whole health district population and SARS-CoV-2-positive individuals.

Age, gender, and residential postcode were collected for the whole health district population (≥18 years) from the 2021 Australian Census [16] and all SARS-CoV-2 PCR-positive individuals in the health district (≥18 years) from the Notifiable Conditions Records for Epidemiology and Surveillance (NCRES).

#### Hospitalised patients.

All SARS-CoV-2 PCR-positive individuals ≥18 years, with their first admission for a COVID-19-associated diagnostic code, were included. Admissions to other hospitals in the health district and readmissions >24 hours following discharge were excluded. Readmissions within 24 hours were considered part of the first presentations and included. Electronic medical records were manually reviewed to confirm eligibility and de-identified data was collected and stored in the Redcap database (REDCap, Vanderbilt University). Data collected included age at admission, sex, language (English or language other than English), home postcode, whether Aboriginal or Torres Strait Islander, number of COVID-19 vaccinations (0–2), current pregnancy and trimester, known co-morbidities, COVID-19 severity scores based on WHO classification [17], length of stay, requirement for supplementary oxygen, ICU admission, and critical complications of SARS-CoV-2 infection during hospitalisation.

### Data analysis

Data cleaning and descriptive analysis were performed in RStudio (version 4.3.2; tidyverse and dplyr packages). Figures were produced using the ggplot2 and sjPlot packages. Categorical variables were represented via counts and percentages, and continuous variables by median and interquartile ranges (IQR). Socioeconomic disadvantage was defined using Socioeconomic Indexes for Areas (SEIFA 2021, Australian Bureau of Statistics) [18]. SEIFA is constructed using various census data points, including educational attainment, employment status, English proficiency, occupation categories, marital status, health conditions, internet connectivity, and annual income. This index ranks different geographic areas, based on their relative socioeconomic advantage or disadvantage. In this study, postcodes for each patient were used to allocate an index of relative socioeconomic disadvantage deciles, ranging from 1 (the most disadvantaged) to 10 (the least disadvantaged areas). To permit statistical comparisons and address the skewed population distribution, hospitalised individuals were compared by grouping into three groups: SEIFA Decile 1, Deciles 2–5, and Deciles 6–10.

### Composite outcome for hospitalised patients

Due to the low mortality rate, a composite outcome was developed to increase statistical power and to assess predictors of severe COVID-19 outcomes. This composite outcome was defined as in-hospital death or any other in-hospital critical complications associated with COVID-19, including extracorporeal membrane oxygenation, invasive ventilation, inotropic support, renal replacement therapy, non-invasive ventilation, thromboembolic complications, and myocardial infarction. These critical complications were included based on the WHO clinical progression scale for hospitalised individuals [19].

### Statistical analysis

Gender distribution and median ages between populations were compared using the two-proportion z-test and the Mann-Whitney U-test. Proportions of individuals in each SEIFA decile were compared using a Chi-squared test with Bonferroni correction. Initial association analysis of continuous non-parametric variables was performed by the Kruskal-Wallis tests followed by Dunn’s test, while for categorical variables, the Chi-square of independence along with Bonferroni correction was used.

### Model development

A generalised linear model was created to determine the association between predictors and the composite outcome. Variables were screened using univariate regression analysis, and those with moderate association with the composite outcome (p < 0.25) or those with known clinical importance were included in the preliminary model. Backward elimination was performed where variables and their interactions with significant evidence of association with the response were included (p < 0.05). Any main effects associated with included interactions were kept in the model regardless of the p-value. Confounder variables initially eliminated were re-tested in the final model. SEIFA and vaccination status were considered to be confounders *a priori*. Model reliability was assessed using a leave-one-out analysis. All modelling and analyses were done using R version 4.3.2.

## Results

Between January 23, 2020, and December 31, 2021, the total adult population in the WSLHD catchment was 797,343. In this time interval, 50,906 (6.38%) were SARS-CoV-2 PCR positive; of these individuals, 2332 (4.58%) were admitted to hospital. Among admitted patients, 1,962 met the inclusion criteria of the study. Patients were excluded if they were <18 years (n = 9), admitted to another hospital (n = 24) and hospitalised at home (n = 337). There were 73 in-hospital deaths, although deaths at home, in another hospital or during subsequent admission were not included.

### Demographic and socioeconomic disadvantage distributions

**Fig 1:**
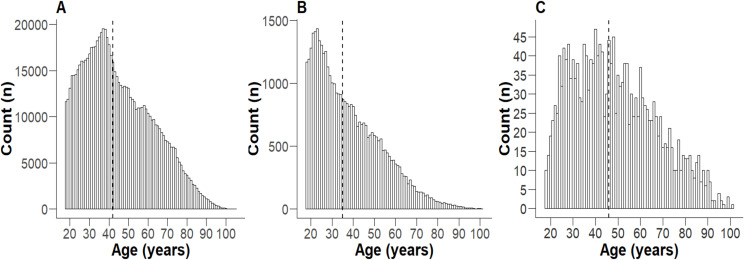
Age distribution of individuals **≥ 18 in 2020-2021.** A) the whole health district population, B) all SARS CoV-2 PCR-positive individuals in the health district, and C) all patients hospitalised with COVID-19. The dashed vertical line represents the median.

**Fig 2:**
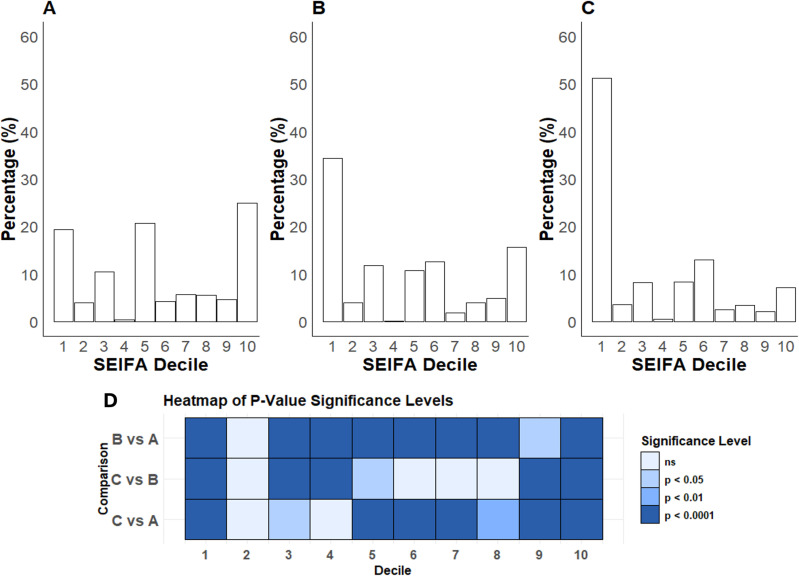
Population distribution (%) of socioeconomic disadvantage. A) the whole health district population. B) all SARS-CoV-2 PCR-positive individuals in the health district. C) all patients hospitalised for COVID-19. D) Heatmap illustrating the significant levels of differences for pairwise comparisons; ns = not significant.

Hospitalised SARS-CoV-2-positive patients were significantly older, with a greater proportion of females than SARS-CoV-2 PCR-positive individuals and the entire health district population (Fig 1, Table 1). The population of the health district had a relatively even distribution of socioeconomic disadvantage (Fig 2A). In contrast, both SARS-CoV-2 PCR-positive individuals and hospitalised patients demonstrated a significant skew towards the first decile (most disadvantaged) (Fig 2B, 2C). Notably, over half of those hospitalised were from the postcodes with the greatest socioeconomic disadvantage, while <10% were from postcodes with the least socioeconomic disadvantage (Fig 2C).

**Table 1:** Age and gender distribution of each population.

Total		Age (years)		Sex	
	Total	Median	IQR	Male (%)	Female (%)
WSLHD Population	797,343	42	31-58	49.9	50.1
WSLHD SARS CoV-2 PCR-positive individuals	50,906	35^b^	25-48	50.6^a^	49.4^a^
Hospitalised Patients	1,962	46^b^^,^ ^c^	33-61	46.5^a^^,^ ^c^	53.5^a^^,^ ^c^

^a^: Compared to health district population (p < 0.01),

^b^: Compared to health district population (p < 0.001),

^c^: Compared to health district SARS CoV-2 cases (p < 0.001).

### Characteristics of hospitalised patients stratified by socioeconomic disadvantage

Demographic and clinical characteristics for hospitalised COVID-19 patients (n = 1,934, 28 excluded due to unknown postcode) in each SEIFA group are shown in Table 2. Hospital admissions were 53.2% female, had a median (IQR) age of 46 (33–61) years; 5.6% were pregnant (10.6% of all hospitalised women), and most spoke English at home (66.7%). Thirty-eight (2.0%) identified as Aboriginal or Torres Strait Islander. Most patients (56.3%) were unvaccinated, with mild COVID-19 severity at admission (58.1%). Most patients (72%) had at least one comorbidity (S1 Table in S1 File), and 21% had four or more comorbidities. The median length of stay was 3 (1–9) days; 1091 (56.4%) patients required supplementary oxygen, 272 (14.1%) were admitted to the ICU, and 73 (3.8%) inpatients died. The composite outcome was present in 222 (11.5%) hospitalised patients (S2 Table in S1 File).

**Table 2:** Demographic and clinical characteristics of hospitalised patients by SEIFA.

SEIFA	Total n = 1934	Decile 1 n = 990	Decile2–5 n = 399	Decile 6–10 n = 545	P-Value
Age (years)					0.07
Median	46	46	47	47	
IQR	33-61	32-62	34-61	35-61	
Sex					0.55
Male	905 (46.8)	459 (46.4)	196 (49.1)	250 (45.9)	
Female	1029 (53.2)	531 (53.6)	203 (50.9)	295 (54.1)	
Pregnancy Trimester (Females only)					0.52
1st	17 (1.7)	9 (1.7)	6 (3.00)	2 (0.7)	
2nd	23 (2.2)	12 (2.3)	7 (3.5)	4 (1.4)	
3rd	69 (6.7)	44 (8.3)	9 (4.4)	16 (5.4)	
Language					<0.0001
English	1289 (66.7)	558 (56.4)	297 (74.4)	434 (79.6)	
Non-English	630 (32.6)	423 (42.7)	98 (24.6)	109 (20.0)	
Not stated	15 (0.8)	9 (0.9)	4 (1.0)	2 (0.4)	
Ethnicity					0.015
Aboriginal or Torres State Islander	38 (2.0)	12 (1.2)	14 (4.0)	12 (2.2)	
Others	1791 (92.6)	929 (93.8)	360 (90.2)	502 (92.1)	
Not stated	105 (5.4)	49 (4.9)	25 (6.3)	31(5.7)	
Vaccine dose					<0.0001
0	1089 (56.3)	604(61.0)	214 (53.6)	271 (49.7)	
1	350 (18.1)	175 (17.7)	90 (22.6)	85 (15.6)	
2	326 (16.9)	125 (12.6)	67 (16.8)	134 (24.6)	
Not stated	169 (8.7)	86(8.7)	28(7.0)	55(10.0)	
Severity					0.06
Asymptomatic	76 (3.9)	33 (3.3)	21 (5.3)	22 (4.0)	
Mild	1124 (58.1)	564 (57.0)	224 (56.1)	336 (61.7)	
Moderate	496 (25.7)	275 (27.8)	97 (24.3)	124 (22.8)	
Severe	154 (8.0)	79 (8.0)	33 (8.3)	42 (7.7)	
Critical	26 (1.3)	16 (1.6)	7 (1.8)	3 (0.6)	
Critical w/ Sepsis	28 (1.4)	8 (0.8)	8 (2.0)	12 (2.2)	
Not stated	30 (1.6)	15(1.5)	9(2.3)	6(1.1)	
Comorbidities					0.51
0	542 (28.0)	289 (29.2)	99 (24.8)	154 (28.3)	
1	448 (23.2)	229 (23.1)	88 (22.1)	131 (24.0)	
2	330 (17.0)	169 (17.1)	80 (20.1)	81 (14.9)	
3	207 (10.7)	102 (10.3)	42 (10.5)	63 (11.6)	
4 or more	407 (21.1)	201 (20.3)	90 (22.6)	116 (21.3)	
Length of Stay (Days)					0.26
Median	3	3	4	4	
IQR	1-9	1-8	1-9.5	1-10	
Supplementary O2	1091(56.4)	596 (60.2)	231(57.9)	264 (48.4)	<0.001
ICU Admission	272 (14.1)	135 (13.6)	64 (16.0)	73 (13.4)	0.41
Mortality	73 (3.8)	35 (3.5)	16 (4.0)	22 (4.0)	0.85
Composite outcome	222 (11.5)	106 (10.7)	51 (12.8)	65 (11.9)	0.49

Data expressed as n (%) of group total).

Those hospitalised were characterised by high levels of relative socioeconomic disadvantage, with 990 of 1934 patients (51.2%) in Decile 1, 399 (20.6%) in Decile 2–5, and 545 (28.2%) in Decile 6–10. Compared to those in the higher SEIFA deciles, those in Decile 1 were more likely to speak a language other than English (42.7%, p < 0.0001), more likely to be unvaccinated (61%, p < 0.0001), more likely to require supplemental oxygen (p < 0.001) and less likely to be Aboriginal or Torres Strait Islander (p = 0.015). Other characteristics were similar, although those in the lowest SEIFA decile tended to be younger (p = 0.06), less critically ill with sepsis at admission and more likely to have moderate COVID-19 (p = 0.06). The proportion of patients with the composite outcome was not different between the SEIFA deciles (p = 0.49). The distribution of the categorical predictor variables by the composite outcome is shown in the S3 Table in S1 File.

### Multivariate regression model

For univariate analysis, SEIFA (tercile), age (age quartile), sex, comorbidities count, ethnicity group, primary language, vaccination status, and COVID severity as a binary variable (asymptomatic/mild/moderate vs severe/critical/critical with sepsis) were included (S3 Table in S1 File).

**Fig 3:**
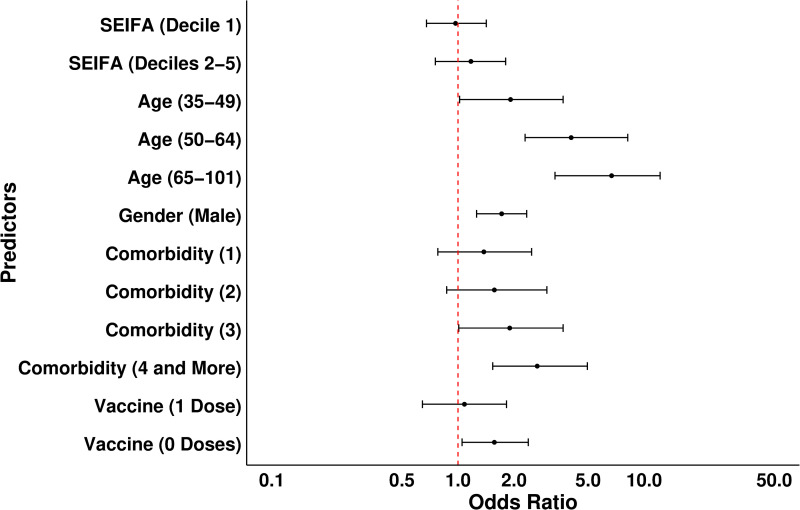
Odds ratios and 95% CI for variables predicting composite outcome. The dashed red line indicates the odd ratio of 1 (reference category meaning no effect).

After the backward elimination process, age, sex, and comorbidities were retained as significant predictors in the final model. SEIFA and vaccination were included due to being confounders *a priori* (S4 Table in S1 File). The results of the multivariate regression model are summarised in Table 3 and Fig 3. Increasing age was the biggest risk for the composite outcome. Patients aged 35–49 years had approximately twice the risk compared to younger patients, with the risk increasing to 4 times for those 50–64 and 7 times for those aged 65 years and older.

**Table 3:** Multivariate regression model for the composite outcome.

Predictors	OR	95% CI	p-value
SEIFA			0.64
Deciles 6–10 (ref)	–	–	
Deciles 2–5	1.17	0.75, 1.80	
Decile 1	0.97	0.68, 1.42	
Age quartile (years)			<0.0001
18-34 (ref)	–	–	
5-49	1.92	1.02, 3.67	
50-64	4.05	2.30, 8.17	
65-101	6.96	3.32, 14.57	
Sex			<0.001
Female (ref)	–	–	
Male	1.72	1.26, 2.42	
Comorbidities			0.006
0 (ref)	–	–	
1	1.38	0.78, 2.44	
2	1.57	0.87, 2.83	
3	1.90	1.01, 3.56	
4 and more	2.67	1.54, 4.63	
Vaccine dose			0.040
2 (ref)	–	–	
1	1.08	0.64, 1.81	
0	1.57	1.05, 2.38	

Men were nearly twice as likely to develop the composite outcome as women. Compared to individuals with no comorbidities, patients with three comorbidities had approximately a 2-fold increase in risk, and risk increased with the increasing number of comorbidities. Unvaccinated patients had a 1.5-fold increased likelihood of developing the composite outcome. Notably, the risk of composite outcome did not increase with socioeconomic disadvantage (Table 3, Fig 3).

## Discussion

This unique, retrospective analysis of a population in a local health district with broad demographic and socioeconomic diversity at the epicentre of the COVID-19 pandemic between Jan 2020 and December 2021 demonstrates that individuals from areas associated with the greatest socioeconomic disadvantage were more likely to be infected with SARS-CoV-2 and more likely to be hospitalised. Remarkably, over half of the hospitalised cohort in this period were from postcodes with the greatest socioeconomic disadvantage. However, in contrast to our hypothesis, once hospitalised for COVID-19, residence in a socioeconomically disadvantaged postcode had no bearing on the likelihood of severe COVID-19 outcomes (a composite outcome of death and other critical complications). Indeed, despite the high prevalence of socioeconomically disadvantaged patients, the in-hospital mortality was notably low, supporting the lack of impact of socio-economic disadvantage on the in-hospital mortality rate. The other risk factors for severe outcomes of COVID-19 identified in our study were age, male sex, coexisting disease and being unvaccinated. This is in keeping with already identified risks [20–23], confirming the similarity of our study population with other populations studied globally and the generalisability of our findings.

Higher infection rates in populations with the greatest socioeconomic disadvantage is a worldwide phenomenon reported in other pandemics such as influenza [24]. These increased rates in most disadvantaged areas are likely multifactorial and associated with higher density living conditions, language barriers, lower health literacy, limited access to primary care, and occupational exposure [25,26], all of which are important factors in the healthcare equity framework [27]. The infected population were also younger and more likely to be male, which was likely a consequence of both living arrangements and employment requirements. Higher rates of infection among working-age individuals, particularly in those unable to work from home, have been reported in COVID-19 [28] and other infectious pandemics [29]. According to the 2021 census data, approximately 64% of males and 57% of females in the local health district are employed. The top three industry sectors for males are construction (14.1%), transport, postal and warehousing (9.7%), and manufacturing (9.2%) and for females, health care and social assistance (22.5%), education and training (12.8%), and retail trade (10.8%) [30]. Thus, within this health district, many individuals, particularly males, work in occupations where it is impossible to work from home, likely contributing to a higher infection rate in younger, working-age males.

Hospitalised individuals were older, more likely to be female, and more than half were from postcodes associated with the greatest socioeconomic disadvantage. Increased hospitalisation among older individuals is likely due to the known association between increased age and more severe illness [20]. The higher admission rate amongst women, despite lower overall infections, could be driven by pregnancy, which is a risk for higher hospital admission [31].

The overall mortality rate was notably low in this study, with in-hospital death of 3.8%. Early international studies reported higher hospital mortality rates, and around 11–50% of hospitalised patients died due to COVID-19 [32–34]. Several factors are likely to have impacted mortality rates, including the SARS-Cov2 variant, improving therapies and vaccination rates, and the presence of travel and contact restrictions ensuring sufficient hospital capacity. Many patients were hospitalised at the peak of the Delta variant (July 2021-December 2021), which is associated with higher mortality [35]; however, the overall mortality remained low. Hospitalisation primarily occurred at a later timing in the worldwide pandemic, when therapies such as dexamethasone [36], antiviral medications [37], antithrombotic medication [38], and immune modulators [39] were established, and clear protocols in place for COVID-19 clinical management, likely reducing mortality. All patients received universal, standardised, protocolised care in the hospital, with a living document used to guide clinical management that was constantly updated and may have contributed to the lower in-hospital mortality. Vaccination within the study region began in late February 2021, and although not high in this cohort, likely further reduced mortality rates [23], particularly as there was a phased approach that targeted frontline workers and priority groups at high risk such as Aboriginal and Torres Strait Islanders, with mass vaccination centres opening in the health district in August 2021, an important approach to health equity by addressing a system of power [27]. In addition, Public Health Orders in the region began in March 2020, including school closures and restrictions on movement to contain the number of infected individuals and prevent the health system from becoming overwhelmed by insufficient capacity [40]. The large number of asymptomatic and mild patients hospitalised may be due in part to admission at a time when COVID-19 was a novel infection, but also likely due to social factors such as the inability to isolate at home.

The majority of hospitalised patients were from areas with the greatest socioeconomic disadvantage. These patients had a higher proportion of non-English speakers, lower vaccination rates, an increased need for oxygen, and a trend towards more moderate COVID-19 severity. However, they were not at an increased risk of severe in-hospital outcomes in either the univariate analysis or after controlling for age, comorbidity, sex and vaccination. These findings are in contrast with international reports of COVID-19 clinical outcomes that greater socioeconomic disadvantage is positively associated with poor clinical outcomes [3,6]. They also contrast with reports up to April 2022 from the Australian Institute of Health and Welfare of increased mortality in those from postcodes with greater socioeconomic disadvantage [13].

Australia has universal health coverage, with the aim of ensuring that all people have access to a full range of healthcare services without financial disadvantage [41]. The reasons socioeconomic disadvantage did not result in more severe COVID-19 outcomes in this study are unclear. However, this may relate to the health care setting of a tertiary referral teaching hospital with highly specialised services and access to standardised, carefully protocolised, publicly funded and timely care that was universally available, with no capacity constraints as a consequence of travel and contact restrictions. This lack of capacity constraints within the hospital, including access to the latest medication, meant that the hospital system was resilient and able to withstand the short-term shock of the COVID-19 pandemic [42].

Apart from vaccination rates and oxygen requirements, other factors such as age, sex, and comorbidity, which may have impacted in-hospital outcomes, were not different, although those with greater socioeconomic disadvantage tended to be younger. This suggests that universal access to appropriate clinical care may overcome the risk of severe in-hospital outcomes in COVID-19 in the most socioeconomically disadvantaged patients [43].

Internationally, socioeconomic disadvantage has been associated with increased in-hospital mortality. In Scotland, area-level socio-economic disadvantage in patients admitted to an intensive care unit was associated with 3 times higher 30-day mortality after adjusting for age, sex, and severity of illness [44]. Similarly, a single Italian hospital reported in multivariate analysis lower educational levels predicted in-hospital mortality, in addition to age and comorbidities [45]. Another study in the United States involving four hospitals with an integrated health system showed that patients from neighbourhoods with lower median income were significantly more likely to require mechanical ventilation and intensive care admission after adjusting for multiple confounders [6]. In a single tertiary care hospital in New Orleans, disproportionate COVID-19 hospitalisations were reported in Black patients, particularly Black women. Mechanical ventilation was more likely with obesity and increasing comorbidities, and mortality was increased in those with a race other than Black or White, age over 65, and increasing comorbidities [46]. Similar to our findings, this study failed to find any impact of socioeconomic factors of insurance status and employment on either mortality or mechanical ventilation. In contrast to our low in-hospital mortality rate of 3.8%, this study reported a higher mortality rate of 13.8% and primarily focused on the effects of race rather than socioeconomic disadvantage. However, the absence of the impact of socio-economic disadvantage on these outcomes was attributed to high-quality care.

The findings of this study can be generalised to inform the management of future pandemics, particularly the importance of dealing with health inequities [47]. Implementing targeted health strategies to increase the vaccination rate and reduce infection rates amongst the most disadvantaged population is critical, and is likely to reduce hospitalisation rates. In addition, this study demonstrates the importance of universally available, high-quality hospital care to ensure equitable outcomes for all populations. Finally, addressing inequality both locally and globally is essential for future pandemic preparedness.

## Strengths and limitations

This retrospective study benefits from being in a healthcare district that included postcodes encompassing all levels of socioeconomic disadvantage, allowing for consideration of demographic, clinical, and socioeconomic factors that may have impacted outcomes. During the chosen time period, the majority of COVID-19 patients in the health district requiring admission were admitted to a single hospital until mid-August 2021, representing nearly all hospitalised patients, with only 1% admitted elsewhere. There was no variation in patient care, as admissions were predominantly to a single well-resourced hospital where patient care was universal, accessible, protocolised and standardised regardless of socioeconomic background. It is possible that a less well-resourced hospital may have had different outcomes.

The limitations of the study are that patients transferred to other hospitals requiring intensive care due to capacity constraints were not included, nor were those who did not present to the hospital or died at home or in an aged care facility, and this may have resulted in an underestimation of the overall mortality rate, in addition to preservation of hospital resources. The study focused exclusively on in-hospital care, and patients hospitalised at home were excluded as they were managed under different protocols. Selection of patients for hospitalisation at home was based on the severity of illness and suitable home environment, which would have favoured those with less disadvantage. Despite this, there were no differences between the groups in terms of COVID-19 severity, although those with the greatest socioeconomic disadvantage had a trend towards more severe disease and were more likely to require oxygen, suggesting that clinical need was the primary reason for admission.

The SEIFA index is an area-based indicator; while it is used as a proxy measure of individual level, many people are likely to be misclassified, limiting some of the interpretation. Additionally, SEIFA groupings may have masked some distinctions between the higher deciles. However, as the majority of patients admitted were from areas with the greatest socioeconomic disadvantage, and the population in the other deciles was mostly less than 10% of the total hospitalised patients, this limitation is unlikely to have greatly altered the outcome.

Although univariate analysis showed no obvious association between socioeconomic disadvantage and in-hospital mortality due to COVID-19, there were insufficient events to analyse the mortality rate in a statistical regression model. Therefore, a composite outcome of mortality and other critical complications of COVID-19 was chosen as the main outcome. Other outcomes, such as length of stay, were considered likely influenced by factors other than clinical need, such as the ability to isolate at home if other family members were not infected, the lack of secure housing, or poor health literacy.

Finally, it is important to note that the findings of this study should be interpreted within the limitations of an observational retrospective design, evolving admission policies and the specific context of the local healthcare setting.

## Conclusion

In the first two years of the COVID-19 pandemic, while the increased prevalence of infection and hospitalisation in the whole district population was associated with the greatest socioeconomic disadvantage, this study finds that once hospitalised, the likelihood of severe in-hospital outcomes was not significantly greater in the disadvantaged group. We infer that well-resourced, universally accessible, publicly funded, standardised and protocolised medical care effectively contributed to improved equity of outcomes in the face of the greatest public health crisis in recent history.

## Supporting information

S1 File **S1 Table.** Summary of comorbidities in COVID-19 hospitalised patients (N = 1962). **S2 Table**. Summary of critical complications (excluding death) in COVID-19 hospitalised patients (N = 1962). **S3 Table.** Distribution of the categorical predictor variables by composite outcome in COVID-19 hospitalised patients (N = 1962). **S4 Table.** Univariate regression analysis for composite outcome in COVID-19 hospitalised patients.
(DOCX)
