# Supplementary material for: Universal health care delivery mitigates socioeconomic-related risk for adverse outcomes in hospitalised patients: Lessons from the COVID-19 pandemic in Australia
Source: PLoS One. 2025 May 14;20(5):e0322780. doi: 10.1371/journal.pone.0322780 (PMC12077700; doi:10.1371/journal.pone.0322780)
Supplement: S1 File — S1 Table. Summary of comorbidities in COVID-19 hospitalised patients (N = 1962). S2 Table. Summary of critical complications (excluding death) in COVID-19 hospitalised patients (N = 1962). S3 Table. Distribution of the categorical predictor variables by composite outcome in COVID-19 hospitalised patients (N = 1962). S4 Table. Univariate regression analysis for composite outcome in COVID-19 hospitalised patients. (DOCX) [file pone.0322780.s001.docx]

**Supplementary material**

**Universal health care delivery mitigates socioeconomic-related risk for adverse outcomes in hospitalised patients: Lessons from the COVID-19 pandemic in Australia**

Fahimeh Faqihi, Rita Perri, Jimmy Chien, Jin-Gun Cho, Stephen Milne, Shopna Bag, Nicole Gilroy, John Wheatley, Kristina Kairaitis

**S1 Table: Summary of comorbidities in COVID-19 hospitalised patients (N = 1962).**

| Comorbidities |  | Count (%) |
| --- | --- | --- |
| Hypertension | | 513 (13.74) |
| Obesity | | 403 (10.80) |
| Diabetes | | 346 (9.27) |
| Heart Disease (excl. Hypertension) | | 273 (7.31) |
| Psychiatric Disease | | 227 (6.08) |
| Asthma | | 196 (5.25) |
| Chronic Neurological Disease | | 148 (3.96) |
| Chronic Pulmonary Disease | | 108 (2.89) |
| Chronic Kidney Disease | | 88 (2.36) |
| Malignant Neoplasm | | 83 (2.22) |
| Chronic Haematological Disease | | 60 (1.61) |
| Dementia | | 35 (0.94) |
| Immunocompromised | | 35 (0.94) |
| Mild Liver Disease | | 30 (0.80) |
| Rheumatological Disease | | 20 (0.54) |
| Moderate/Severe Liver Disease | | 9 (0.24) |
| Tuberculosis | | 7 (0.19) |
| AIDS/HIV | | 3 (0.08) |
| Asplenia | | 3 (0.08) |

**S2 Table: Summary of critical complications (excluding death) in COVID-19 hospitalised patients (N = 1962).**

| Complications | Count (Percentage) |
| --- | --- |
| ECMO | 10 (0.5) |
| Invasive Ventilation | 82 (4.2) |
| Inotropic Support | 54 (2.8) |
| Renal Replacement Therapy | 21 (1.1) |
| Venous Thromboembolism | 40 (2.0) |
| Deep Vein Thrombosis | 32 (1.6) |
| Non-Invasive Ventilation | 101 (5.2) |
| Pulmonary Embolism | 21 (1.1) |
| Arterial Thromboembolism | 22 (1.1) |
| Myocardial Infarction | 26 (1.3) |

**S3 Table: Distribution of the categorical predictor variables by composite outcome in COVID-19 hospitalised patients (N = 1962).**

| Predictor | Value | No Outcome  n(%) | Outcome  n(%) |
| --- | --- | --- | --- |
| Total |  | 1,740 (88.7) | 222 (11.3) |
| SEIFA Tercile | Decile1 | 884 (89.3) | 106 (10.7) |
|  | Deciles 2-5 | 348 (87.2) | 51 (12.8) |
|  | Deciles 6-10 | 480 (88.1) | 65 (11.9) |
| Age Quartile | 18-34 | 514 (97.2) | 15 (2.8) |
|  | 35-49 | 536 (93.5) | 37 (6.5) |
|  | 50-64 | 380 (84.6) | 69 (15.4) |
|  | 65-101 | 310 (75.4) | 101 (24.6) |
| Ethnic Group | ATSI | 33 (86.8) | 5 (13.2) |
|  | Non-ATSI | 1,616 (89.0) | 200 (11.0) |
| Primary Language | English | 1,162 (88.6) | 150 (11.4) |
|  | Other | 578 (88.9) | 72 (11.1) |
| Comorbidity | No comorbidity | 530 (95.8) | 23 (4.2) |
|  | One comorbidity | 424 (92.8) | 33 (7.2) |
|  | Two comorbidities | 295 (89.1) | 36 (10.9) |
|  | Three comorbidities | 176 (83.8) | 34 (16.2) |
|  | Four or more comorbidities | 315 (76.6) | 96 (23.4) |
| Gender | Male | 779 (85.3) | 134 (14.7) |
|  | Female | 961 (91.6) | 88 (8.4) |
| Vaccination Status | Fully Vaccinated | 289 (87.0) | 43 (13.0) |
|  | Partially Vaccinated | 320 (90.7) | 33 (9.3) |
|  | Unvaccinated | 978 (88.7) | 125 (11.3) |
| Covid Severity | Asymptomatic/Mild/Moderate | 1,617 (93.9) | 105 (6.1) |
|  | Severe/Critical/Critical with Sepsis | 96 (45.9) | 113 (54.1) |

**S4 Table: Univariate regression analysis for composite outcome in COVID-19 hospitalised patients**

| **Predictor** | **OR** | **Lower Bound** | **Upper Bound** | **P-value** |
| --- | --- | --- | --- | --- |
| **SEIFA Tertile** |  |  |  |  |
| Decile1 | 0.88 | 0.64 | 1.23 | 0.46 |
| Decile2-5 | 1.08 | 0.72 | 1.60 | 0.69 |
| Decile6-10 | 0.13 | 0.10 | 0.17 | <0.0001 |
| **Age Quartile** |  |  |  |  |
| 18-34 | 0.02 | 0.01 | 0.04 | <0.0001 |
| 35-49 | 2.37 | 1.31 | 4.49 | <0.001 |
| 50-64 | 6.22 | 3.60 | 11.4 | <0.0001 |
| 65-101 | 11.2 | 6.57 | 20.3 | <0.0001 |
| **Sex** |  |  |  |  |
| Female | 0.09 | 0.07 | 0.11 | <0.0001 |
| Male | 1.88 | 1.42 | 2.50 | <0.0001 |
| **Primary Language** |  |  |  |  |
| English | 0.12 | 0.10 | 0.15 | <0.0001 |
| Other | 0.96 | 0.71 | 1.30 | 0.81 |
| **Comorbidities** |  |  |  |  |
| 0 | 0.04 | 0.02 | 0.06 | <0.0001 |
| 1 | 1.79 | 1.04 | 3.14 | 0.036 |
| 2 | 2.81 | 1.65 | 4.90 | <0.001 |
| 3 | 4.45 | 2.57 | 7.85 | <0.0001 |
| 4 or more | 7.02 | 4.44 | 11.5 | <0.0001 |
| **Vaccination dose** |  |  |  |  |
| 2 | 0.14 | 0.10 | 0.20 | <0.0001 |
| 1 | 0.69 | 0.42 | 1.12 | 0.13 |
| 0 | 0.85 | 0.59 | 1.26 | 0.42 |
